# Body image in head and neck cancer patients treated with radiotherapy: the impact of surgical procedures

**DOI:** 10.1186/s12955-017-0740-7

**Published:** 2017-08-23

**Authors:** Tsung-Min Hung, Ching-Rong Lin, Yu-Chun Chi, Chien-Yu Lin, Eric Yen-Chao Chen, Chung-Jan Kang, Shiang-Fu Huang, Yeong-Yuh Juang, Chun-Yu Huang, Joseph Tung-Chieh Chang

**Affiliations:** 1 Department of Radiation Oncology, Chang Gung Memorial Hospital at Linkou, No.5 Fu-Shin Street, Kwei-Shan, Taoyuan, Taiwan; 2 Department of Radiation Oncology, Xiamen Chang Gung Hospital, Xiamen City, Fujian Province China; 3 School of Nursing, College of Medicine, Chang Gung University, Taoyuan, Taiwan; 4 Department of Psychology, Chung Yuan Christian University, 200 Chung Pei Road, Chung Li District, Taoyuan City, 32023 Taiwan, Republic of China; 5 Department of Radiation Oncology, Chang Gung Memorial Hospital at Keelung, Keelung, Taiwan; 6 Department of Otorhinolaryngology, Head and Neck Surgery, Chang Gung Memorial Hospital at Linkou, Taoyuan, Taiwan; 7 Department of Psychiatry, Chang Gung Memorial Hospital at Linkou, Taoyuan, Taiwan

## Abstract

**Background:**

In this study, we aimed to investigate the impact of surgical procedures on the body image of head and neck cancer patients treated with radiotherapy and with or without radical surgery.

**Methods:**

A cross-sectional survey of 150 patients with head and neck cancer was conducted. Sixty patients had nasopharyngeal cancer treated with definitive radiotherapy without surgery, and 90 patients had oral cavity cancer treated with radical surgery plus adjuvant radiotherapy. All participants completed a 10-item Body Image Scale (BIS) questionnaire to assess body image dissatisfaction. Among all patients, the socio-demographic and clinical variables were age, gender, partnership, education, employment, and radical surgery. In surgically-treated patients, the clinical variables were facial skin sacrificed, mouth angle sacrificed, glossectomy, maxillectomy, and mandibulectomy. ANOVAs, t-tests, and multiple regressions were used to evaluate the relationships between these variables and BIS results.

**Results:**

In all patients, radical surgery was the strongest independent predictor of BIS scores. Surgically-treated patients had significantly worse BIS scores than the patients without surgery. In surgically-treated patients, facial skin sacrificed, mouth angle sacrificed, maxillectomy, and mandibulectomy were significantly associated with body image. According to multivariable analyses, inferior maxillectomy and segmental mandibulectomy were independent prognosticators of a poor BIS score in surgically-treated patients.

**Conclusion:**

Radical surgery for head and neck cancer patients has a significant impact on their body image, especially for those undergoing facial bone destructive surgery.

## Background

Body image is a subjective concept that includes self-perceptions, thoughts and feelings, with links to social factors, and cancer and its treatment often negatively influence body image [1]. Patients with head and neck cancer (HNC) usually experience significant issues with body image because of visible disfigurement and organ dysfunction from both the disease itself and the treatment [2]. Gamba et al. [3] reported that HNC patients with extensive disfigurement after surgery had significantly higher impact on their self-image. Besides, nearly one-fifth persons stated that the disadvantages of treatment outweighed the advantages, and 30% of the study group fell that the difficulties they faced were “too harsh” [3]. Fingeret et al. [4] reported that individuals with speech and eating concerns had increased body image dissatisfaction compared to those without such concerns. The available reports regarding body image concerns in HNC primarily focus on the experiences of surgically treated patients [2–5].

However, in addition to surgery, radiation therapy is also a common treatment modality for patients with various types of HNC. In patients with nasopharyngeal cancer (NPC), radiation therapy serves as the major treatment, and no surgery is typically necessary. Furthermore, in patients with oral cavity cancer (OCC), who usually undergo radical surgery as the primary treatment, radiotherapy plays a role in the adjuvant setting for those with a high risk of disease recurrence.

Due to the paucity of body image research on HNC patients treated with radiation therapy, we initiated this cross-sectional study to investigate body image outcomes in irradiated HNC patients. We were interested in whether the different treatment methods used (surgical vs. non-surgical treatment) among patients with various types of HNC influenced body image. Moreover, we wished to assess the impacts of different surgical procedures on the body image of surgically treated irradiated HNC patients.

## Methods

### Design and participants

The Institutional Review Board of the hospital approved this cross-sectional study. Applying G*Power 3.17 to estimate the sample size of our study, when setting effect size = 0.5, α = 0.05, and power = 0.8, the required sample size is 134. Finally, we enrolled 150 HNC patients treated with radiotherapy in a three-to-two proportion of surgically treated to non-surgically treated patients. The inclusion criteria were as follows: (1) HNC patients who were diagnosed by an oncologist and had completed the entire course of treatment; (2) HNC patients older than 20 years of age without cognitive deficits who could communicate with words or speech; HNC patients who (3) had no active HNC disease while participating in this study; (4) had no disfigurement throughout the entire body (such as amputation or facial burns) before the diagnosis of HNC; (5) had no diagnosis of major psychological disease (such as schizophrenia); and (6) agreed to participate in this research.

All participants were recruited through the follow-up outpatient clinics of the department of radiation oncology at one medical center hospital in Taiwan. A total of 166 HNC patients were asked to participate in this study, but 16 patients (9.6%) refused, while 150 patients agreed and were enrolled. Every participant signed the informed consent form before beginning the survey. For making sure that the sample size of this study is adequate, we conducted the power analysis by G*Power 3.17, and the power of our study was 85% when setting effect size = 0.5 and α = 0.05.

### Variables and measures

The participant provided the socio-demographic data (age, gender, partnership, education, and employment status). The Body Image Scale (BIS) self-report questionnaire was completed by each participant [5]. At least one investigator or assistant was available for the participant to answer any questions while completing the questionnaire. The face of the participant was observed by the investigator to determine whether any defects of the facial skin or mouth angle existed.

The investigator collected the clinical variables (cancer type, treatment modality, time after irradiation, and surgical procedures) by reviewing medical chart records. The items regarding surgical procedures included operational techniques, such as whether facial skin was sacrificed, whether mouth angle was preserved, whether a glossectomy was performed, whether an inferior maxillectomy was performed, and whether a mandibulectomy was performed. The extent of the dissection for a glossectomy was divided into partial glossectomy (less or equal to hemiglossectomy) and total glossectomy (total or nearly total glossectomy). The methods for a mandibulectomy were marginal mandibulectomy and segmental mandibulectomy. All of the clinical information collected was checked a second time by at least one oncologist.

The BIS, a 10-item questionnaire, was used to assess body image. This instrument was initially validated in patients with breast cancer [6] and was subsequently proved to have adequate internal consistency and high correlations with other body image measures in OCC patients [7]. The traditional Chinese version of the BIS used in this study was the version translated by Tsui [8]. Higher scores on the BIS indicated an increased body image disturbance. The internal reliability (Cronbach’s alpha) of the BIS in this study was 0.94. Applying the Kolmogorov-Smirnov Z test to check the normal distribution of our study, both the 60 NPC patients without surgery (*p* = 0.08 > 0.05) and the 90 surgically-treated OCC patients (*p* = .12 > 0.05) conformed normal distributions of BIS.

### Statistical analysis

A descriptive analysis of the socio-demographic and clinical variables was conducted. ANOVAs, t-tests, and multiple regressions were used to evaluate the relationships between these variables and BIS scores. Univariable analyses were performed using t-tests and ANOVAs. For the multivariable analysis, a linear regression model with backward selection was used to test the independent influence of the socio-demographic and clinical variables on body image. A *p*-value of less than 0.05 was considered to be statistically significant. All statistical analyses were performed using SPSS 21.0 (IBM-SPSS Inc., Chicago, IL, USA).

## Results

Among the 150 HNC patients enrolled in this study, 60 patients were treated for NPC with definitive radiotherapy but no surgery, and 90 patients were treated for OCC with radical surgery plus adjuvant radiotherapy. The socio-demographic and clinical variables among all patients are summarized in Table 1.

**Table 1:** Patient characteristics (*n* = 150)

Characteristic	Number (%)
Age (years)	
Mean ± SD	50.85 ± 8.44
Range	29–72
Gender	
Male	128 (85.3)
Female	22 (14.7)
Partnered	
Yes	118 (78.7)
No	32 (21.3)
Education level	
Primary school	27 (18.0)
Junior high school	43 (28.7)
Senior high school	48 (32.0)
University and above	32 (21.3)
Employment status	
No job	38 (25.3)
Part-time job	55 (36.7)
Full-time job	57 (38.0)
Cancer type	
Nasopharyngeal carcinoma	60 (40.0)
Oral cavity cancer	90 (60.0)
Treatment modality	
RT alone	10 (6.7)
CCRT	50 (33.3)
Surgery + RT	34 (22.7)
Surgery + CCRT	56 (37.3)
Time after irradiation	
Within 2 years	66 (44.0)
More than 2 years	84 (56.0)

*Abbreviations: RT* Radiotherapy, *CCRT* Concurrent chemoradiation

The cancer subtypes among the 90 patients with OCC were as follows: 32 patients had tongue cancer, 29 had buccal cancer, 9 had gingival cancer, 5 had retromolar cancer, one had floor of the mouth cancer, and 14 patients had two or more types of OCC. The patients with tongue cancer and floor of the mouth cancer underwent glossectomies as the primary surgery, and the patients with other subtypes of OCC were treated by the wide local excision of the buccal, gingival, or retromolar tumors. Forty-two patients had surgery with facial skin sacrificed, and 26 patients did not have the mouth angle preserved. Twenty-four patients had operations with inferior maxillectomy. Twenty-seven patients were treated by surgery with segmental mandibulectomy, and 24 were treated with marginal mandibulectomy. There were high correlations between facial bone destructive surgery (maxillectomy or mandibulectomy) and facial skin damage (surgery with facial skin or mouth angle sacrificed). In all, 75% of the patients who had facial skin or mouth angle sacrificed also underwent inferior maxillectomy or segmental mandibulectomy. All of the OCC patients had a reconstructive operation after the radical surgery.

The results of the univariable analysis demonstrated that the surgically-treated patients had a significantly worse body image than those without surgery for each item of the BIS (see Table 2).

**Table 2:** The mean score on each item of the BIS by radical surgery or not

Scale item (range)	Radical surgery (*n* = 90) Mean score (SD)	No surgery (*n* = 60) Mean score (SD)	*p-*value
1 Self-conscious (0–3)	1.63 (0.91)	0.68 (0.73)	<0.001
2 Less physically attractive (0–3)	1.28 (0.92)	0.62 (0.78)	<0.001
3 Dissatisfied with appearance (0–3)	1.43 (1.04)	0.53 (0.77)	<0.001
4 Less feminine/masculine (0–3)	0.84 (0.95)	0.27 (0.45)	<0.001
5 Difficult to see self naked (0–3)	1.08 (0.97)	0.33 (0.57)	<0.001
6 Less sexually attractive (0–3)	1.16 (1.03)	0.50 (0.77)	<0.001
7 Avoid people (0–3)	1.14 (0.99)	0.35 (0.71)	<0.001
8 Body less whole (0–3)	1.30 (0.95)	0.65 (0.94)	<0.001
9 Dissatisfied with body (0–3)	1.19 (1.00)	0.65 (0.80)	<0.001
10 Dissatisfied with scar (0–3)BIS total score (0–30)	1.44 (1.00) 12.50 (7.95)	0.42 (0.70)5.00 (4.92)	<0.001<0.001

*Abbreviations: BIS* Body image scale

The results of the univariable analysis between BIS scores and socio-demographic or clinical variables are summarized in Table 3. We found that age, gender, partnership and time after irradiation are not significant factors for body image outcome in the univariable analysis. Educational level and employment status significantly influenced the BIS scores in all patients, but not in subgroup analysis (treated with surgery or without surgery).

**Table 3:** Univariable analysis between the mean score on the BIS and the socio-demographic or clinical variables

Variable	All (*n* = 150)			No surgery (*n* = 60)			Radical surgery (*n* = 90)		
	*n*	Mean (SD)	*p* (post-hoc)	*n*	Mean (SD)	*p*	*n*	Mean (SD)	*p*
Age (years)									
≦50	69	9.5 (8.1)	0.992	31	5.2 (5.6)	0.717	38	13.0 (8.2)	0.613
> 50	81	9.5 (7.6)		29	4.8 (4.1)		52	12.1 (7.8)	
Gender									
Male	128	9.7 (8.0)	0.361	45	4.8 (4.9)	0.510	83	12.4 (8.0)	0.845
Female	22	8.1 (6.8)		15	5.7 (5.0)		7	13.1 (7.8)	
Partnered									
Yes	118	9.9 (7.9)	0.241	45	5.2 (5.1)	0.699	73	12.8 (7.9)	0.506
No	32	8.1 (7.4)		15	4.4 (4.3)		17	11.3 (8.2)	
Education level									
Primary school (a)	27	13.0 (9.0)	0.001	8	5.4 (4.7)	0.142	19	16.2 (8.5)	0.061
Junior high school (b)	43	9.5 (7.3)	(a, c > d)	17	6.6 (5.6)		26	11.4 (7.8)	
Senior high school (c)	48	10.3 (7.7)		16	5.5 (5.5)		32	12.7 (7.6)	
University and above (d)	32	5.3 (5.7)		19	2.9 (3.3)		13	8.8 (6.8)	
Employment status									
No job (a)	38	12.6 (8.5)	0.012	5	6.4 (8.0)	0.675	33	13.5 (8.3)	0.488
Part-time job (b)	55	9.0 (7.6)	(a > c)	24	4.4 (4.2)		31	12.6 (7.7)	
Full-time job (c)	57	7.9 (7.0)		31	5.2 (5.0)		26	11.0 (7.9)	
Treatment modality									
RT alone (a)	10	5.6 (6.7)	<0.001	10	5.6 (6.7)	0.676			0.532
CCRT (b)	50	4.9 (4.6)	(d > a)	50	4.9 (4.6)				
Surgery + RT (c)	34	11.8 (8.2)	(c, d > b)				34	11.8 (8.2)	
Surgery + CCRT (d)	56	12.9 (7.9)					56	12.9 (7.9)	
Time after irradiation									
Within 2 years	66	10.0 (8.2)	0.476	21	4.7 (4.8)	0.876	45	12.5 (8.3)	0.984
More than 2 years	84	9.1 (7.5)		39	5.2 (4.9)		45	12.5 (7.7)	

*Abbreviations: BIS* Body image scale, *NPC* Nasopharyngeal carcinoma, *OCC* Oral cavity cancer, *RT* Radiotherapy, *CCRT* Concurrent chemoradiation

**Fig. 1:**
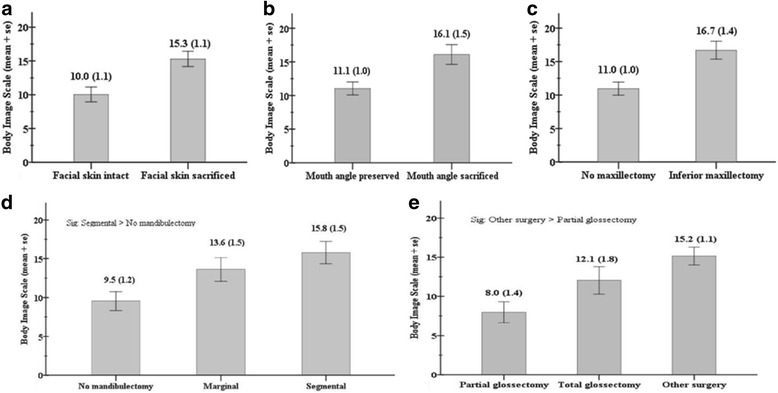
Body image dissatisfaction of the surgically treated patients based on different surgical procedures: (**a**) surgically-treated patients with intact facial skin (*n* = 48) or facial skin sacrificed (*n* = 42), *p* = 0.001; (**b**) surgically-treated patients with mouth angle preserved (*n* = 64) or mouth angle sacrificed (*n* = 26), *p* = 0.006; (**c**) surgically-treated patients with inferior maxillectomy (*n* = 24) or no maxillectomy (*n* = 66), *p* = 0.002; (**d**) surgically-treated patients with marginal mandibulectomy (Marginal, *n* = 24), segmental mandibulectomy (Segmental, *n* = 27), or no mandibulectomy (*n* = 39), *p* = 0.004; (**e**) surgically-treated patients with partial glossectomy (*n* = 25), total glossectomy (*n* = 19), or other surgery (*n* = 46, underwent wide local excision of buccal/gingival/retromolar tumors), *p* = 0.001

In surgically-treated patients, the relationship between different surgical procedures and BIS results revealed that the patients with facial skin sacrificed, mouth angle sacrificed, inferior maxillectomy, and segmental mandibulectomy had a significantly worse body image (Fig. 1).

Using a multivariable analysis, we found that radical surgery and education were the independent factors in predicting BIS scores in all patients. The R^2^ and adjusted R^2^, the variance of the model, is 0.26 and 0.25, respectively. In surgically-treated patients, partial glossectomy, inferior maxillectomy and segmental mandibulectomy were the independent prognosticators of body image. The R^2^ and adjusted R^2^, the variance of the model, is 0.23 and 0.20, respectively. The results of the multivariable analysis are summarized in Table 4.

**Table 4:** Multiple linear regression models for the BIS

Multiple linear regression model^a^ for all patients (*n* = 150)				
	Reduced model			
	Estimate	*p*	95% CI	
			Lower	Upper
Intercept	6.15	<0.001	4.22	8.08
Radical surgery (ref: no surgery)	6.88	<0.001	4.60	9.15
University and above (ref: primary school)	- 3.63	0.009	- 6.36	- 0.91
Multiple linear regression model^b^ for the surgically treated patients (*n* = 90)				
	Reduced model			
	Estimate	*p*	95% CI	
			Lower	Upper
Intercept	11.46	<0.001	9.03	13.89
Partial glossectomy (ref: other surgery^c^)	- 4.45	0.014	- 7.99	- 0.91
Segmental (ref: no mandibulectomy)	3.92	0.020	0.63	7.21
Maxillectomy (ref: no maxillectomy)	4.13	0.023	0.57	7.69

*Abbreviations: BIS* Body image scale; Segmental, segmental mandibulectomy; Maxillectomy, inferior maxillectomy

^a^Excluded variables: age, gender, partnered, employment.; R^2^ = 0.26, adjusted R^2^ = 0.25

^b^Excluded variables: age, gender, partnered, education, employment, facial skin sacrificed, mouth angle sacrificed.; R^2^ = 0.23, adjusted R^2^ = 0.20

^c^Other surgery: wide local excision of buccal/gingival/retromolar tumors

**Fig. 2:**
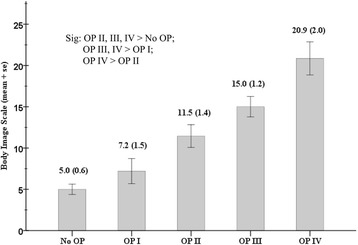
Body image dissatisfaction of all patients (*n* = 150) divided by radical surgery and the three surgical procedures*: No OP (*n* = 60, the patients who did not receive radical surgery), OP I (*n* = 20, surgically-treated patients who had partial glossectomy but without inferior maxillectomy or segmental mandibulectomy), OP II (*n* = 31, surgically-treated patients who had one of the three surgical procedures*), OP III (*n* = 32, surgically-treated patients who had two of the three surgical procedures*), and OP IV (*n* = 7, surgically-treated patients who had all three surgical procedures*), *p* < 0.001. *Three surgical procedures were inferior maxillectomy, segmental mandibulectomy, and not partial glossectomy (total glossectomy or wide local excision of buccal/gingival/retromolar tumors)

Using the three surgical procedures with independently significant associations with the BIS, we further divided the surgically-treated patients into four groups: patients with none, one, two, or three of the three surgical procedures. The body image outcomes of the patients without surgery and these four groups of surgically-treated patients were compared, and the results are presented in Fig. 2.

## Discussion

From the results of the current study, we discovered that radical surgery was the strongest independent predictor of BIS scores among all patients. The non-surgically treated patients had a significantly better body image outcome than the surgically treated patients for each item of the BIS. This finding revealed that radical surgery for HNC patients indeed has a significant impact on body image. Several previous studies ﻿[9, 10] also demonstrated that body image and facial appearance satisfaction in HNC patients are worse after surgery than before surgery.

Furthermore, in the surgically treated patients, three surgical procedures (partial glossectomy, inferior maxillectomy, and segmental mandibulectomy) were found to be independent prognosticators of body image. The patients treated with partial glossectomy but without inferior maxillectomy and segmental mandibulectomy had the best body image outcomes among the surgically treated patients. For the three surgical procedures, inferior maxillectomy, segmental mandibulectomy, and total glossectomy or wide local excision for buccal/gingival/retromolar tumors (not partial glossectomy), the more of these procedures performed on the surgically treated patients, the greater the body image dissatisfaction. The patients who underwent a total glossectomy or wide local excision for buccal/gingival/retromolar tumors, in addition to inferior maxillectomy and segmental mandibulectomy, had the worst body image. There were high correlations between facial bone destructive surgery and operations resulting in facial skin damage; 75% of the patients with the facial skin or mouth angle sacrificed also underwent inferior maxillectomy or segmental mandibulectomy. This association might be the reason that the sacrifice of facial skin or mouth angle was a significant factor for BIS scores in the univariable analysis but not in the multivariable analysis.

Body image is affected by both disfigurement and dysfunction. Fingeret et al. [4] reported that body image dissatisfaction was significantly higher among HNC patients with both speech and eating concerns. In our study, the reasons that the patients treated with inferior maxillectomy or segmental mandibulectomy had worse body image outcomes might include both facial bone destruction (disfigurement) and eating/speech problems (dysfunction).

In a recent review article, Fingeret et al. [11] provided potential indicators of body image difficulties in adult cancer patients, which would help in referring patients who require psychosocial care. In our study, we found that radical surgery for HNC patients was a significant factor in body image dissatisfaction, and the three surgical procedures (partial glossectomy, inferior maxillectomy, and segmental mandibulectomy) were independent prognosticators of body image outcomes in surgically treated patients. These findings could be used to select those HNC patients suitable for the development of psychosocial interventions. Semple et al. [12] reported that evidence for psychosocial interventions for HNC patients is limited based on a review of the published literature by these authors. Future research to provide evidence-based interventions for this population is required.

There are several limitations in the present study, including the cross-sectional design, heterogeneity of the time elapsed after irradiation, and the inclusion of patients with only NPC or OCC. Furthermore, the participants in this study were all treated at one medical center hospital with aggressive treatments, which may limit the application of our findings. Nevertheless, our report is noteworthy because this study provides a comparison of body image outcomes among different treatment methods of head and neck cancer patients including NPC and OCC. This study provides an important finding that different surgical procedures have a strong impact on BIS scores in head and neck cancer patients.

## Conclusions

In conclusion, this study found that radical surgery for HNC patients has a significant impact on body image dissatisfaction. Furthermore, the surgically treated patients who had the surgical procedures with facial bone destruction have an even worse body image outcome. These findings could be used to select patients with HNC to receive psychosocial interventions targeting body image disturbance.

## Abbreviations

BIS: Body image scale
CCRT: Concurrent chemo-radiation
HNC: Head and neck cancer
NPC: Nasopharyngeal carcinoma
OCC: Oral cavity cancer
RT: Radiotherapy
